# Emergency Department Presentations of Chronic Kidney Disease in a Tertiary Hospital of Nepal: An Observational Study

**DOI:** 10.31729/jnma.9116

**Published:** 2025-06-30

**Authors:** Ram Prasad Neupane, Ramesh Kumar Maharjan, Bipsana Shrestha, Bibek Poudel, Rasmila Dandekhya, Tirtha Man Shrestha, Shasank Chitrakar, Anga Raj Dulal, Arju Malla, Amisha Silwal, Sagun Karki, Manju Pokhrel, Shubham Kumar Thakur, Abhinandan Panthee, Sagar Kumar Jha, Anish Sah, Bishnu Bhujel

**Affiliations:** 1 Department of Emergency Medicine, Maharajgunj Medical Campus, Institute of Medicine, Maharajgunj, Kathmandu, Nepal; 2 Health Directorate, Ministry of Health, Bagmati Province, Hetauda, Nepal; 3 Department of Medicine, Palpa hospital, Tansen, Palpa; 4 Department of General Practice, Maharajgunj Medical Campus, Institute of Medicine, Maharajgunj, Kathmandu, Nepal; 5 Paropakar Nursing Campus, Kupondole, Lalitpur, Nepal.

**Keywords:** *chronic kidney disease*, *emergency*, *end stage renal disease*

## Abstract

**Introduction:**

Progressive chronic kidney disease is associated with complications like hypertension, anemia, mineral bone disorder, metabolic acidosis and electrolyte disorders which lead to higher morbidity, mortality and poorer quality of life. Available estimates of chronic kidney disease cases and those receiving treatment suggests an existing gap in care. Therefore, this study aims to estimate the prevalence of chronic kidney disease and its complications in the emergency department of Tribhuvan University Teaching Hospital.

**Methods:**

A cross-sectional descriptive study was done. The participants were chronic kidney disease patients with complications visiting the Emergency Department of Maharajgunj Medical Campus. Census sampling technique was used to enroll all chronic kidney disease patients with complications meeting inclusion criteria, visiting the Emergency Department from 11^th^ October 2023 to 19^th^ September 2024. Ethical approval was obtained from the institutional review committee of the Institute of Medicine, Maharajgunj with the reference number 509(6-11)E2/081/082. Distribution check and descriptive analysis was done using STATA.

**Results:**

The prevalence of chronic kidney disease was found to be 2.5%. The most common complications were anemia 399 (53.69%) and hypertensive crisis 396 (53.29%). Least common complications among CKD patients was sepsis 8 (1.08%). Several other complications like arrhythmia, nephropathy, hypoglycemia, obstructive uropathy, pericardial effusion, urinary tract infection, pleural effusion, pneumonia were observed in 50 (6.73%) of the CKD patients. Death as an outcome was reported in 9 (1.2%) of total patients.

**Conclusions:**

Chronic kidney disease proportion in emergency departments is high, with anemia and hypertension being common complications. Targeted interventions might include establishing hemodialysis units and increasing staff awareness.

## INTRODUCTION

Progressive chronic kidney disease (CKD) is associated with complications like hypertension, anemia, mineral bone disorder, metabolic acidosis and electrolyte disorders. These complications lead to higher morbidity, mortality and poorer quality of life.^1^ Emergency hospital visits are almost six times more by a patient diagnosed with end stage renal disease (ESRD) compared to an average adult.^2^

Despite these devastating consequences, the mere estimation of incidence of ESRD is difficult to ascertain because there is no Renal Registry in Nepal.^3^ The available predictions are based on global and Indian incidence estimates. In 2016, 2,900 people were estimated to be suffering from ESRD in Nepal, however, only 1,975 people received dialysis. This suggests an existing gap in care for a large group of ESRD patients.^4^

Therefore, this study aims to estimate the prevalence of CKD in the emergency department of Tribhuvan University Teaching Hospital (TUTH), along with the complications presented by those patients.

## METHODS

This is a cross-sectional descriptive study. STROBE reporting guidelines checklists were considered. The participants of this study were CKD patients presenting with complications. The study was conducted at the Emergency department of Tribhuwan University Teaching Hospital (TUTH), which is a high-volume patient flow health facility that serves a diverse patient population from diverse regions of Nepal, including a significant number of individuals presenting with CKD and its complications.

The sampling technique used was census sampling technique which enrolled all CKD patients with complications visiting the Emergency Department who met the inclusion criteria of the study. The study duration lasted from 11^th^ October, 2023 to 19^th^ September, 2024. The study proposal was reviewed and approved by the institutional review committee (IRC) of the Institute of Medicine, Maharajgunj with the ethical approval granted under the reference number 509(6-11 )E2/081/082. All procedures were followed in accordance with the ethical standards established by IRC. Confidentiality and privacy of information of patients were ensured. Formal permission was taken from the respective hospital administration to access and utilize the medical record data for research purposes.

Inclusion criteria for the study were adults ≥18 years with confirmed Chronic Kidney Disease (CKD) and presenting to ED with CKD-related emergencies. Patients with acute kidney injury without prior CKD were excluded from the study. Further, those CKD-patients with complications who had incomplete medical records were also excluded from the study. Data was extracted from the emergency census and book of the emergency department. Midas software was used for extracting the lab reports of the patients. The data captured key clinical and demographic information of patients with CKD including information about the presence of various complications. The variables included in the dataset are as follows: patient's age, sex, provisional diagnosis, date of admission, complications presented in the emergency department, presence of comorbidity and clinical outcome such as discharged, referral to other healthcare institutions, leave against medical advice (LAMA), discharge on patient request (DOPR), shift to ICU, mortality in the emergency department. The appropriate variables were selected during analysis to reflect the prevalence of CKD, its complications and mortality in CKD patients following their presentation to the emergency department.

Data was then finally recorded in the kobotoolbox platform. Recorded data was exported in Microsoft Excel 2016 and subsequently imported into STATA version 14.0 for analysis. Distribution check was done which showed that the distribution was approximately normal as shown by an approximately symmetric histogram. Descriptive statistical analysis was done to report frequencies and percentages to present categorical variables. For prevalence, 95% CI was also reported.

## RESULTS

Out of a total of 743 patients who presented to the emergency department during the specified study period, the mean age of the patients was 50.5±17.6 years. There were 347 (46.71%) pateints who fell within the age group of 40 to 64 years. There were 216 (29.06%) patients who were under 40 years of age, while 180 (24.23%) patients were aged 65 years and above. In terms of gender distribution, there were 482 (64.88%) male and 261 (35.12%) females (Table 1).

**Table 1:** Sociodemographic variables (n=743)

Variables	Frequency (%)
Age in years (mean±SD)	50.5±17.6
Below 40	216(29.06)
40 to 64	347(46.71)
65 and above	180(24.23)
Sex	
Male	482(64.88)
Female	261(35.12)

The overall prevalence of chronic kidney disease (CKD) among all individuals who visited the emergency department was found to be 2.50% (3.20-2.60 at 95% Confidence Interval). Among the patients included in this study, 9 (1.21%) patients were documented as having death as the clinical outcome at the emergency department visit.

Complications observed in this study included anemia in 399 (53.69%) patients, hypertensive crisis in 396 (53.29%) patients and pulmonary edema in 112 (15.08%) patients. Hyperglycemia was reported in 60 (8.08%) patients, electrolyte imbalance in 50 (6.73%) patients, metabolic acidosis in 34 (4.58%) patients, and uremic gastritis in 29 (3.90%) patients. Uremic encephalopathy was diagnosed in 18 (2.42%) patients, and sepsis was noted in 8 (1.08%) patients (Table 2).

Additional complications including arrhythmia, nephropathy, hypoglycemia, obstructive uropathy, pericardial effusion, urinary tract infection, pleural effusion, and pneumonia were grouped together and observed in 50 (6.73%). Furthermore, 394 (53.03%), presented with multiple complications simultaneously. Comorbid conditions such as diabetes mellitus and hypertension were present in 395 (53.16%).

**Table 2:** Complications in Chronic Kidney Disease patients (n=743).

Complications	Frequency (%)
Anemia	399 (53.69)
Hypertensive crisis	396 (53.29)
Hyperglycemia	60 (8.08)
Electrolyte imbalance	50 (6.73)
Metabolic acidosis	34 (4.58)
Uremic gastritis	29 (3.90)
Pulmonary edema	112 (15.08)
Uremic encephalopathy	18 (2.42)
Sepsis	8 (1.08)
Others	50 (6.73)
Comorbidity	395 (53.16)

## DISCUSSION

The findings of our study showed hypertensive crisis as one of the most common (53.29%) while sepsis as one of the least common (1.08%) complications in CKD patients. However, one of the studies done in emergency centers of Southwest Nigeria reported hypertensive crisis (12.9%) as well as sepsis (16.8%) and uraemia (21.3%) as the most common presentations in CKD patients.^5^ Hypertension has been one of the most damaging complications of CKD. It enhances the acceleration of the progressive decline in kidney functions and increase in cardiovascular diseases (CVD), leading to increased mortality in both cases.^6^ Although improved blood pressure would help patients directly, both detection as well as control of high blood pressure are mostly suboptimal.^7^ One of the feasible goal to control hypertension as complications in CKD patients would be to assure antihypertensive medicine adherence because such medicines and easily available and affordable in lower and middle income countries (LMICs).^8^ Further, a study done in TUTH itself in 2018 showed that 6.7% of the CKD patients presented with sepsis which is higher than in the present study.^9^

Our findings showed anemia as one of the most common (53.69%) complications in CKD patients. A study done in Canada also showed anemia (14.7%) as one of the most common chronic complications of CKD.^10^ Evidences suggest the most important and specific cause of anemia in CKD patients is reduced erythropoietin synthesis. Additionally, anemia in CKD can be due to various other mechanisms like iron deficiency, folate deficiency, vitamin B12 deficiency, gastrointestinal bleeding, shortened red blood cell survival, systemic inflammation, and severe hyperparathyroidism.^11,12^ For the longest time, the major treatment methods used to treat anemia in CKD has been the use of erythropoiesis-stimulating agents (ESA) and iron supplementation via oral or intravenous route. These treatment methods should also be combined with the management of aforementioned reversible causes of anemia.^13^

The international society of nephrology in 2017 has also included hypertension and anaemia in the list of readily defined and quantified complications of CKD along with others such as mineral bone disorder, volume overload, electrolytes, and acid-base abnormalities. It also mentioned some other less well- defined complications of CKD like anorexia, nausea, fatigue, pruritus, cachexia, and sexual dysfunction. These less well-defined complications are often associated with advanced stages of CKD.^1^

Our findings showed 1.2% deaths among all CKD patients presenting in the emergency department. In contrast to this, the study conducted in the emergency department of TUTH in 2018 reported higher mortality (7.7%) in CKD patients.^9^ The study done in Southwest Nigeria comparatively reported higher mortality (8.9%) in CKD patients.^5^ CKD is one of the prominent emerging causes of mortality and morbidity in the 21^st^ century. In 2017, an estimated 843.6 million individuals were affected by CKD worldwide. This increase in the number of CKD cases is partly due to an increase in risk factors of CKD such as obesity and diabetes mellitus.^14^ Therefore, it is vital to identify, monitor and treat CKD rigorously. Along with that, implementing preventive and therapeutic measures to address CKD worldwide is equally important.^15^

The mean age of CKD patients in this study is 50.5±17.6 years. A prior study in TUTH's emergency department in 2018 reported a similar mean age of 45.6±17.2 years, indicating little demographic shift in CKD patient profiles over the past five years.^9^ There are diverse relations of CKD, age of patients and mortality. One of the studies reported that the adjusted relative risk of mortality with CKD was higher (3.50, 95% CI 2.554.81) in younger patients (aged 18-54 years) and lower (1.35, 95% CI 1.23-1.48) in older people (aged 75 years and above). However, the impact of excess deaths was more in patients older than 75 years (27.2 per 1000 person-years) compared to younger people (9.4 per 1000 person-years).^16^

Based on the professional experiences of authors of this study working in the emergency department, newly appointed medical officers and residents often face challenges in managing patients with CKD, particularly in the treatment of acute complications requiring urgent hemodialysis. To address this gap, the authors emphasize the need to familiarize new officers and residents with the local burden of CKD from the outset of their appointment. The authors recommend the establishment of an emergency- dedicated hemodialysis unit within or adjacent to the emergency department, which would enable timely and appropriate intervention for CKD patients presenting with acute complications.

Further, there is an urgent need of running dedicated emergency dialysis for CKD patients admitted in the emergency department. A cohort study done in a tertiary care center created a novel pathway for identification and facilitation of earlier treatment of CKD patients called fast track dialysis (FTD). This FTD was especially targeted for those who need urgent and routine hemodialysis. The study showed that there was reduction in length of hospital stay, hospital charges, and arrival time to hemodialysis through the use of FTD.^2^ Hence, dedicated dialysis is crucial in the emergency department of any hospital. The Ministry of Health, Government of Nepal has offered free lifetime hemodialysis since 2016.^4^ However, a large number of impoverished patients who need hemodialysis still do not receive treatment. This might be due to other indirect costs such as transportation costs to hemodialysis centers, lost wages, cost for food, cost of diagnostic investigations or that of medicines.^3^

There are several strengths of this study. One of the major strengths of our study is the census sampling technique which eliminates the sampling bias by including all CKD patients of the emergency department during study period who meet inclusion criteria of the study. Further, a year long data collection ensures inclusion of seasonal variations of CKD-related emergencies. Excluding those patients with incomplete information might have helped in maintaining data accuracy. Although this study has many strengths, it also has few limitations. One of those limitations is while census sampling reduces sampling bias, there might still exist selection bias. This is because excluding patients with incomplete records might disproportionately exclude more severe or complex cases, limiting the information on complications presentation in CKD patients. Additionally, since this study is based on the data from an emergency department of a single tertiary hospital, the results may not be generalized to the other health care settings or other areas, reducing the external validity.

## CONCLUSIONS

The study reveals a high proportion of chronic kidney disease (CKD) in emergency departments, with anemia and hypertension being the most common complications with low mortality rates.
